# Supplementary material for: scAAVengr, a transcriptome-based pipeline for quantitative ranking of engineered AAVs with single-cell resolution
Source: eLife. 2021 Oct 19;10:e64175. doi: 10.7554/eLife.64175 (PMC8612735; doi:10.7554/eLife.64175)
Supplement: Supplementary file 1. — AAV selection rounds in canines 2b and 5b were repeated selections of the previous rounds, which did not result in the amplification of AAV variants. [file elife-64175-supp1.docx]

**Supplementary File 1. Table with Summary of injections performed in dogs and primates**. AAV selection rounds in canines 2b and 5b were repeated selections of the previous rounds, which did not result in the amplification of AAV variants.

| **Round of DE selection** | **Species/ ID/**  **Gender** | **DOB** | **Age at injection** | **Virus injected** | **Amount virus injected/eye** | **Notes** |
| --- | --- | --- | --- | --- | --- | --- |
| 1 | Canine  I-430  (F) | 9/16/11 | 11 months | Prepacked Shuffle, AAV2-7mer, EP2 and Loopswap libraries | ~4E+13 vg total, 1E+13 vg each library | No adverse events |
| 2a | Canine  I-429  (F) | 9/16/11 | 14 months | Recovered variants from round 1 | ~1E+13 vg | No variants recovered; no immune response noted |
| 2b | Canine  I-428  (M) | 8/20/11 | 17 months | Recovered variants from round 1 (repeated) | ~1.6E+12 vg | No adverse events (Repeat of previous round) |
| 3 | Canine  CDJCCL  (F) | 10/5/12 | 12 months | Recovered variants from round 2 | ~6.7E+12 vg | OS uveitis |
| 4 | Canine  CEDCZF  (F) | 4/26/13 | 7 months | Recovered EP PCR variants from round 3, SCHEMA library | ~2.54E+12 vg | Error prone PCR conducted, No adverse events |
| 5a | Canine  CEDCVS  (F) | 4/21/13 | 9 months | Recovered variants from round 4 | ~5E+12 vg | Uveitis, no detachment, retina grossly normal, no variants recovered |
| 5b | Canine  CEJCJY  (F) | 10/11/13 | 13 months | Recovered variants from round 4 (repeated) | ~3E+12 vg | No adverse events (Repeat of previous round) |
| GFP-barcode | Canine  CGBCAN  (M) | 2/1/15 | 8 months | GFP-barcode library | 6E+11 vg each of 7 variants in 250 uL | No adverse events |
| GFP-barcode | Canine  CGBCDI  (M) | 2/5/15 | 8 months | GFP-barcode library | 6E+11 vg each of 7 variants in 250 uL | No adverse events |
| GFP-barcode | Canine  CGBCGS  (M) | 2/7/15 | 8 months | GFP-barcode library | 6E+11 vg each of 7 variants in 250 uL | No adverse events |
|  | Marmoset  M9-17  (F) | 07/01/2012 | 7 years | OD: GFP-barcoded AAV library  OS: GFP-barcoded AAV library | ~1.24E+11 vg in 25 µL | No adverse events  AAV2 Nab titer prior to  injection = 1:2 |
|  | Marmoset  M23-17  (F) | 02/09/2014 | 3 years | OS: GFP-barcoded AAV library | ~1.24E +11 vg in 25 µL | No adverse events  AAV2 Nab titer prior to  injection = 1:2 |
|  | Cynomolgus  Macaque  262-19  (F) | ~2016 (Exact D.O.B. unknown) | ~3 years | OD: GFP-barcoded AAV library OS:   GFP-barcoded AAV library | ~6.46E+11 vg in 130 µL | OD and OS uveitis  AAV2 Nab titer prior to  injection = 1:2 |
|  | Cynomolgus  Macaque  M79-19  (F) | ~2016 (Exact D.O.B. unknown) | ~3 years | OD: K912-scCAG-GFP | ~2.6E+12 vg in 120 µL | No adverse events  AAV2 Nab titer prior to  injection = 1:2 |
|  | Rhesus macaque  M273-18  (M) | 8/20/15 | 4 years | OD: K912-saCas9-guideRNA +  K912-scCAG-GFP | ~1.4E+12 vg in 130 ul, +~3.3E+11 vg in 15 ul, +20 ul PBS | No adverse events  AAV2 Nab titer prior to  injection = 1:2 |
|  | Cynomolgus  Macaque  M80-19  (F) | ~2016 (Exact D.O.B. unknown) | ~3 years | OS: K912-saCas9-guideRNA | ~1.2E+12 vg in 100 µl | No adverse events  AAV2 Nab titer prior to  injection = 1:2 |
