# Supplementary material for: scAAVengr, a transcriptome-based pipeline for quantitative ranking of engineered AAVs with single-cell resolution
Source: eLife. 2021 Oct 19;10:e64175. doi: 10.7554/eLife.64175 (PMC8612735; doi:10.7554/eLife.64175)
Supplement: Supplementary file 2. — A. Friedman’s test was conducted to determine differences across AAV variants. The test was run separately for each cell type as well as total cells combined. Marmoset and cynomolgus macaque samples were both used in the analysis (n = 8). Significant P-values < 0.05 are shown in bold red. S2.1 p-values resulting from a Friedman’s test using percent cells infected as the data points. Significant P-values < 0.05 are shown in bold red. S2.2 p-values resulting from a Friedman’s test using average transcripts per infected cell as the data points. Significant P-values < 0.05 are shown in bold red. [file elife-64175-supp2.docx]

**Supplementary file 2.** **Statistical Analysis.** A. Friedman’s test was conducted to determine differences across AAV variants. The test was run separately for each cell type as well as total cells combined. Marmoset and cynomolgus macaque samples were both used in the analysis (n=8). Significant p-values < 0.05 are shown in bold red.

S2.1 p-values resulting from a Friedman’s test using percent cells infected as the data points. Significant p-values < 0.05 are shown in bold red.

| **Cell type** | **p-value** |
| --- | --- |
| Rod | **2.22E-12** |
| Cone | **1.38E-08** |
| Horizontal Cell | **4.70E-12** |
| Off-Bipolar | **3.04E-12** |
| On-Bipolar | **1.26E-12** |
| Amacrine Cell | **3.81E-14** |
| Microglia | **1.14E-10** |
| Muller Glia | **5.73E-13** |
| Retinal Ganglion Cell | **4.50E-11** |
| Total Cells | **4.01E-15** |

S2.2 p-values resulting from a Friedman’s test using average transcripts per infected cell as the data points. Significant p-values < 0.05 are shown in bold red.

| **Cell type** | **p-value** |
| --- | --- |
| Rod | **5.45E-12** |
| Cone | **1.06E-08** |
| Horizontal Cell | **5.31E-12** |
| Off-Bipolar | **3.66E-12** |
| On-Bipolar | **1.74E-12** |
| Amacrine Cell | **1.89E-13** |
| Microglia | **1.36E-10** |
| Muller Glia | **3.15E-13** |
| Retinal Ganglion Cell | **1.91E-11** |
