# Supplementary material for: scAAVengr, a transcriptome-based pipeline for quantitative ranking of engineered AAVs with single-cell resolution
Source: eLife. 2021 Oct 19;10:e64175. doi: 10.7554/eLife.64175 (PMC8612735; doi:10.7554/eLife.64175)
Supplement: Supplementary file 3. — A one-sided Wilcoxon signed-rank test was used for a pairwise comparison between K912 or NHP26 and the other variants. P-values were corrected using Benjamini-Hochberg correction method. Significant P-values < 0.05 are shown in bold red. S3.1 p-values resulting from a one-sided Wilcoxon signed-rank test using percent cells infected as the data points, comparing NHP12 and other variants. S3.2 p-values resulting from a one-sided Wilcoxon signed-rank test using percent cells infected as the data points, comparing NHP26 and other variants. S3.3 p-values resulting from a one-sided Wilcoxon signed-rank test using average transcripts per infected cell as the data points, comparing K912 and other variants. S3.4 p-values resulting from a one-sided Wilcoxon signed-rank test using average transcripts per infected cell as the data points, comparing NHP26 and other variants. [file elife-64175-supp3.docx]

**Supplementary file 3. p-values from a Wilcoxon signed-rank test.** A one-sided Wilcoxon signed-rank test was used for a pairwise comparison between K912 or NHP26 and the other variants. P-values were corrected using Benjamini-Hochberg correction method. Significant p-values < 0.05 are shown in bold red.

S3.1 p-values resulting from a one-sided Wilcoxon signed-rank test using percent cells infected as the data points, comparing NHP12 and other variants.

|  | K912--  AAV1 | K912--  AAV2 | K912--  AAV2.4YF | K912--  AAV2.  4YFTV | K912--  AAV5 | K912--  AAV8 | K912--  AAV8.2YF | K912-  -AAV9 | K912--  AAV9.2YF | K912--  AAVrh10 | K912--  K91 | K912--  K916 | K912--  K94 | K912--  NHP9 | K912--  NHP26 | K912--  SCH.  NHP9.26 |
| --- | --- | --- | --- | --- | --- | --- | --- | --- | --- | --- | --- | --- | --- | --- | --- | --- |
| Rod | **3.91E-03** | **3.91E-03** | **3.91E-03** | **3.91E-03** | **3.91E-03** | **3.91E-03** | **3.91E-03** | **3.91E-03** | **3.91E-03** | **3.91E-03** | **3.91E-03** | **3.91E-03** | **3.91E-03** | **3.91E-03** | **3.91E-03** | **3.91E-03** |
| Cone | **1.38E-02** | **1.38E-02** | **1.38E-02** | **1.38E-02** | **1.38E-02** | **1.38E-02** | **1.38E-02** | **1.38E-02** | **1.38E-02** | **1.38E-02** | **1.38E-02** | 8.04E-02 | 1.36E-01 | **1.38E-02** | **4.46E-02** | **1.38E-02** |
| HC | **1.29E-02** | **1.29E-02** | **1.29E-02** | **1.29E-02** | **1.29E-02** | **1.29E-02** | **1.29E-02** | **1.29E-02** | **1.29E-02** | **1.29E-02** | **1.29E-02** | **1.29E-02** | 5.40E-02 | **1.29E-02** | 2.92E-02 | **1.29E-02** |
| Off-Bipolar | **1.38E-02** | **1.38E-02** | **1.38E-02** | **1.38E-02** | **1.38E-02** | **1.38E-02** | **1.38E-02** | **1.38E-02** | **1.38E-02** | **1.38E-02** | **1.38E-02** | **3.38E-02** | 5.78E-02 | **1.38E-02** | 7.42E-02 | **1.38E-02** |
| On-Bipolar | **3.91E-03** | **3.91E-03** | **3.91E-03** | **3.91E-03** | **3.91E-03** | **3.91E-03** | **3.91E-03** | **3.91E-03** | **3.91E-03** | **3.91E-03** | **3.91E-03** | **3.91E-03** | **3.91E-03** | **3.91E-03** | **3.91E-03** | **3.91E-03** |
| AC | **4.17E-03** | **4.17E-03** | **4.17E-03** | **4.17E-03** | **4.17E-03** | **4.17E-03** | **4.17E-03** | **4.17E-03** | **4.17E-03** | **4.17E-03** | **4.17E-03** | **4.17E-03** | 1.25E-01 | **4.17E-03** | **4.17E-03** | **4.17E-03** |
| Microglia | **4.17E-03** | **4.17E-03** | **4.17E-03** | **4.17E-03** | **4.17E-03** | **4.17E-03** | **4.17E-03** | **4.17E-03** | **4.17E-03** | **4.17E-03** | **4.17E-03** | **4.17E-03** | **4.17E-03** | **4.17E-03** | **1.17E-02** | **4.17E-03** |
| Müller Glia | **4.17E-03** | **4.17E-03** | **4.17E-03** | **4.17E-03** | **4.17E-03** | **4.17E-03** | **4.17E-03** | **4.17E-03** | **4.17E-03** | **4.17E-03** | **4.17E-03** | **4.17E-03** | **1.95E-02** | **4.17E-03** | **4.17E-03** | **4.17E-03** |
| RGC | **4.17E-03** | **4.17E-03** | **4.17E-03** | **4.17E-03** | **4.17E-03** | **4.17E-03** | **4.17E-03** | **4.17E-03** | **4.17E-03** | **4.17E-03** | **4.17E-03** | **4.17E-03** | **4.17E-03** | **4.17E-03** | **7.81E-03** | **4.17E-03** |
| All Cells | **4.17E-03** | **4.17E-03** | **4.17E-03** | **4.17E-03** | **4.17E-03** | **4.17E-03** | **4.17E-03** | **4.17E-03** | **4.17E-03** | **4.17E-03** | **4.17E-03** | **4.17E-03** | **1.17E-02** | **4.17E-03** | **4.17E-03** | **4.17E-03** |

S3.2 p-values resulting from a one-sided Wilcoxon signed-rank test using percent cells infected as the data points, comparing NHP26 and other variants.

|  | NHP26--  AAV1 | NHP26--  AAV2 | NHP26--  AAV2.  4YF | NHP26--  AAV2.  4YFTV | NHP26--  AAV5 | NHP26--  AAV8 | NHP26--  AAV8.2YF | NHP26-  -AAV9 | NHP26--  AAV9.2YF | NHP26--  AAVrh10 | NHP26--  K91 | NHP26--  K912 | NHP26--  K16 | NHP26--  K94 | NHP26--  NHP9 | NHP26--  SCH.  NHP9.26 |
| --- | --- | --- | --- | --- | --- | --- | --- | --- | --- | --- | --- | --- | --- | --- | --- | --- |
| Rod | **4.81E-03** | **4.81E-03** | **4.81E-03** | **4.81E-03** | **4.81E-03** | **4.81E-03** | **4.81E-03** | **4.81E-03** | **4.81E-03** | **4.81E-03** | **4.81E-03** | 1 | 6.25E-02 | 6.71E-01 | **4.81E-03** | **4.81E-03** |
| Cone | **1.50E-02** | **4.64E-02** | **1.50E-02** | **1.50E-02** | **1.50E-02** | **1.50E-02** | **1.50E-02** | **1.50E-02** | **1.50E-02** | **1.50E-02** | **1.50E-02** | 9.73E-01 | 2.01E-01 | 6.05E-01 | **1.50E-02** | **1.50E-02** |
| HC | **4.81E-03** | **4.81E-03** | **4.81E-03** | **4.81E-03** | **4.81E-03** | **4.81E-03** | **4.81E-03** | **4.81E-03** | **4.81E-03** | **4.81E-03** | **4.81E-03** | 9.81E-01 | 8.30E-01 | 9.62E-01 | **4.81E-03** | **4.81E-03** |
| Off-Bipolar | **4.81E-03** | **4.81E-03** | **4.81E-03** | **4.81E-03** | **4.81E-03** | **4.81E-03** | **4.81E-03** | **4.81E-03** | **4.81E-03** | **4.81E-03** | **4.81E-03** | 9.45E-01 | 1.43E-01 | 5.62E-01 | **4.81E-03** | **4.81E-03** |
| On-Bipolar | **4.81E-03** | **4.81E-03** | **4.81E-03** | **4.81E-03** | **4.81E-03** | **4.81E-03** | **4.81E-03** | **4.81E-03** | **4.81E-03** | **4.81E-03** | **4.81E-03** | 1 | 1.79E-01 | 9.00E-01 | **4.81E-03** | **4.81E-03** |
| AC | **4.81E-03** | **4.81E-03** | **4.81E-03** | **4.81E-03** | **4.81E-03** | **4.81E-03** | **4.81E-03** | **4.81E-03** | **4.81E-03** | **4.81E-03** | **4.81E-03** | 1 | 3.12E-01 | 8.79E-01 | **4.81E-03** | **4.81E-03** |
| Microglia | **1.64E-02** | **1.64E-02** | **2.22E-02** | **2.22E-02** | **1.64E-02** | **1.64E-02** | **1.64E-02** | **1.64E-02** | **1.64E-02** | **1.64E-02** | **1.64E-02** | 9.92E-01 | 8.62E-02 | 8.93E-01 | **1.64E-02** | **1.64E-02** |
| Müller Glia | **4.81E-03** | **4.81E-03** | **4.81E-03** | **4.81E-03** | **4.81E-03** | **4.81E-03** | **4.81E-03** | **4.81E-03** | **4.81E-03** | **4.81E-03** | **4.81E-03** | 1 | 3.12E-01 | 8.63E-01 | **4.81E-03** | **4.81E-03** |
| RGC | **4.81E-03** | **4.81E-03** | **4.81E-03** | **4.81E-03** | **4.81E-03** | **4.81E-03** | **4.81E-03** | **4.81E-03** | **4.81E-03** | **4.81E-03** | **4.81E-03** | 9.96E-01 | 1.79E-01 | 7.75E-01 | **4.81E-03** | **4.81E-03** |
| All Cells | **4.81E-03** | **4.81E-03** | **4.81E-03** | **4.81E-03** | **4.81E-03** | **4.81E-03** | **4.81E-03** | **4.81E-03** | **4.81E-03** | **4.81E-03** | **4.81E-03** | 1 | 2.19E-01 | 8.21E-01 | **4.81E-03** | **4.81E-03** |

S3.3 p-values resulting from a one-sided Wilcoxon signed-rank test using average transcripts per infected cell as the data points, comparing K912 and other variants.

|  | K912--  AAV1 | K912--  AAV2 | K912--  AAV2.4YF | K912--  AAV2.4YFTV | K912--  AAV5 | K912--  AAV8 | K912--  AAV8.2YF | K912-  -AAV9 | K912--  AAV9.2YF | K912--  AAVrh10 | K912--  K91 | K912--  K916 | K912--  K94 | K912--  NHP9 | K912--  NHP26 |
| --- | --- | --- | --- | --- | --- | --- | --- | --- | --- | --- | --- | --- | --- | --- | --- |
| Rod | **4.17E-03** | **4.17E-03** | **4.17E-03** | **4.17E-03** | **4.17E-03** | **4.17E-03** | **4.17E-03** | **4.17E-03** | **4.17E-03** | **4.17E-03** | **4.17E-03** | **4.17E-03** | **1.17E-02** | **4.17E-03** | **4.17E-03** |
| Cone | **1.29E-02** | **1.29E-02** | **1.29E-02** | **1.29E-02** | **1.29E-02** | **1.29E-02** | **1.29E-02** | **1.29E-02** | **1.29E-02** | **1.29E-02** | **1.29E-02** | **1.29E-02** | **1.85E-02** | **1.29E-02** | **1.95E-02** |
| HC | **1.29E-02** | **1.29E-02** | **1.29E-02** | **1.29E-02** | **1.29E-02** | **1.29E-02** | **1.29E-02** | **1.29E-02** | **1.29E-02** | **1.29E-02** | **1.29E-02** | **1**.45E-01 | **1.29E-02** | **1.29E-02** | 3.20E-01 |
| Off-Bipolar | **1.38E-02** | **1.38E-02** | **1.38E-02** | **1.38E-02** | **1.38E-02** | **1.38E-02** | **1.38E-02** | **1.38E-02** | **1.38E-02** | **1.38E-02** | **1.38E-02** | **1.85E-02** | **1.85E-02** | **1.38E-02** | 7.42E-02 |
| On-Bipolar | **4.46E-03** | **4.46E-03** | **4.46E-03** | **4.46E-03** | **4.46E-03** | **4.46E-03** | **4.46E-03** | **4.46E-03** | **4.46E-03** | **4.46E-03** | **4.46E-03** | **1.25E-02** | 1.91E-01 | **4.46E-03** | **4.46E-03** |
| AC | **4.81E-03** | **4.81E-03** | **4.81E-03** | **4.81E-03** | **4.81E-03** | **4.81E-03** | **4.81E-03** | **4.81E-03** | **4.81E-03** | **4.81E-03** | **4.81E-03** | **8.93E-03** | 7.42E-02 | **4.81E-03** | **4.17E-02** |
| Microglia | **4.17E-03** | **4.17E-03** | **4.17E-03** | **4.17E-03** | **4.17E-03** | **4.17E-03** | **4.17E-03** | **4.17E-03** | **4.17E-03** | **4.17E-03** | **4.17E-03** | **4.17E-03** | **4.17E-03** | **4.17E-03** | **2.73E-02** |
| Müller Glia | **4.17E-03** | **4.17E-03** | **4.17E-03** | **4.17E-03** | **4.17E-03** | **4.17E-03** | **4.17E-03** | **4.17E-03** | **4.17E-03** | **4.17E-03** | **4.17E-03** | **4.17E-03** | **7.81E-03** | **4.17E-03** | **4.17E-03** |
| RGC | **3.91E-03** | **3.91E-03** | **3.91E-03** | **3.91E-03** | **3.91E-03** | **3.91E-03** | **3.91E-03** | **3.91E-03** | **3.91E-03** | **3.91E-03** | **3.91E-03** | **3.91E-03** | **3.91E-03** | **3.91E-03** | **3.91E-03** |

S3.4 p-values resulting from a one-sided Wilcoxon signed-rank test using average transcripts per infected cell as the data points, comparing NHP26 and other variants.

|  | NHP26--  AAV1 | NHP26--  AAV2 | NHP26--  AAV2.4YF | NHP26--  AAV2.4YFTV | NHP26--  AAV5 | NHP26--  AAV8 | NHP26--  AAV8.2YF | NHP26-  -AAV9 | NHP26--  AAV9.2YF | NHP26--  AAVrh10 | NHP26--  K91 | NHP26--  K912 | NHP26--  K16 | NHP26--  K94 | NHP26--  NHP9 | NHP26--  SCH.NHP9.26 |
| --- | --- | --- | --- | --- | --- | --- | --- | --- | --- | --- | --- | --- | --- | --- | --- | --- |
| Rod | **5.21E-03** | **5.21E-03** | **1.44E-02** | **5.21E-03** | **5.21E-03** | **5.21E-03** | **5.21E-03** | **5.21E-03** | **5.21E-03** | **5.21E-03** | **5.21E-03** | 1 | **4.46E-02** | 7.75E-01 | **5.21E-03** | **5.21E-03** |
| Cone | **1.38E-02** | **1.38E-02** | **1.38E-02** | **1.38E-02** | **1.38E-02** | **1.38E-02** | **1.38E-02** | **1.38E-02** | **1.38E-02** | **1.38E-02** | **1.38E-02** | 9.88E-01 | **1.38E-02** | 1.88E-01 | **1.98E-02** | **1.38E-02** |
| HC | **4.81E-03** | **4.81E-03** | **4.81E-03** | **4.81E-03** | **4.81E-03** | **4.81E-03** | **4.81E-03** | **4.81E-03** | **4.81E-03** | **4.81E-03** | **4.81E-03** | **7.27E-01** | **4.82E-01** | 5.62E-01 | **4.81E-03** | **4.81E-03** |
| Off-Bipolar | **4.81E-03** | **4.81E-03** | **4.81E-03** | **4.81E-03** | **4.81E-03** | **4.81E-03** | **4.81E-03** | **4.81E-03** | **4.81E-03** | **4.81E-03** | **4.81E-03** | 9.45E-01 | **2.23E-02** | 1.33E-01 | **4.81E-03** | **4.81E-03** |
| On-Bipolar | **4.81E-03** | **4.81E-03** | **4.81E-03** | **4.81E-03** | **4.81E-03** | **4.81E-03** | **4.81E-03** | **4.81E-03** | **4.81E-03** | **4.81E-03** | **4.81E-03** | 1 | 4.24E-01 | 1 | **4.81E-03** | **4.81E-03** |
| AC | **4.81E-03** | **4.81E-03** | **4.81E-03** | **4.81E-03** | **4.81E-03** | **4.81E-03** | **4.81E-03** | **4.81E-03** | **4.81E-03** | **4.81E-03** | **4.81E-03** | 9.73E-01 | 8.48E-02 | 9.00E-01 | **4.17E-03** | **4.81E-03** |
| Microglia | **1.38E-02** | **1.38E-02** | **1.38E-02** | **1.38E-02** | **1.38E-02** | **1.38E-02** | **1.38E-02** | **1.38E-02** | **1.38E-02** | **1.38E-02** | **1.38E-02** | 9.81E-01 | **1.98E-02** | 4.50E-01 | **1.38E-02** | **1.38E-02** |
| Müller Glia | **4.81E-03** | **4.81E-03** | **4.81E-03** | **4.81E-03** | **4.81E-03** | **4.81E-03** | **4.81E-03** | **4.81E-03** | **4.81E-03** | **4.81E-03** | **4.81E-03** | 1 | 2.63E-01 | 6.17E-01 | **4.81E-03** | **4.81E-03** |
| RGC | **4.81E-03** | **4.81E-03** | **4.81E-03** | **4.81E-03** | **4.81E-03** | **4.81E-03** | **4.81E-03** | **4.81E-03** | **4.81E-03** | **4.81E-03** | **4.81E-03** | 1 | 1.79E-01 | 6.17E-01 | **4.81E-03** | **4.81E-03** |
