# Supplementary material for: scAAVengr, a transcriptome-based pipeline for quantitative ranking of engineered AAVs with single-cell resolution
Source: eLife. 2021 Oct 19;10:e64175. doi: 10.7554/eLife.64175 (PMC8612735; doi:10.7554/eLife.64175)
Supplement: Supplementary file 4. [file elife-64175-supp4.docx]

**Supplementary File 4. List of primers used in the study.**

| **Primer** | **Sequence** |
| --- | --- |
| HindIII_F1 | GACGTCAGACGCGGAAGCTTC |
| NotI_R1 | GGTTTATTGATTAACAAGCGGCCG |
| 2-7mer Forward adapter | AATGATACGGCGACCACCGAGATCTACACTCTTTCCCTACACGACGCTCTTCCGATCT**NNNNNTCTACCAACCTCCAGAGAGG** |
| rev_index1 | CAAGCAGAAGACGGCATACGAGAT**CGTGAT**GTGACTGGAGTTCAGACGTGTGCTCTTCCGATCTNNNNNGTTGACATCTGCGGTAGCTG |
| rev_index2 | CAAGCAGAAGACGGCATACGAGAT**ACATCG**GTGACTGGAGTTCAGACGTGTGCTCTTCCGATCTNNNNNGTTGACATCTGCGGTAGCTG |
| rev_index3 | CAAGCAGAAGACGGCATACGAGAT**GCCTAA**GTGACTGGAGTTCAGACGTGTGCTCTTCCGATCTNNNNNGTTGACATCTGCGGTAGCTG |
| rev_index4 | CAAGCAGAAGACGGCATACGAGAT**TGGTCA**GTGACTGGAGTTCAGACGTGTGCTCTTCCGATCTNNNNNGTTGACATCTGCGGTAGCTG |
| rev_index5 | CAAGCAGAAGACGGCATACGAGAT**CACTGT**GTGACTGGAGTTCAGACGTGTGCTCTTCCGATCTNNNNNGTTGACATCTGCGGTAGCTG |
| rev_index6 | CAAGCAGAAGACGGCATACGAGATATTGGCGTGACTGGAGTTCAGACGTGTGCTCTTCCGATCTNNNNNGTTGACATCTGCGGTAGCTG |
| rev_index7 | CAAGCAGAAGACGGCATACGAGAT**GATCTG**GTGACTGGAGTTCAGACGTGTGCTCTTCCGATCTNNNNNGTTGACATCTGCGGTAGCTG |
| rev_index8 | CAAGCAGAAGACGGCATACGAGAT**TCAAGT**GTGACTGGAGTTCAGACGTGTGCTCTTCCGATCTNNNNNGTTGACATCTGCGGTAGCTG |
| rev_index9 | CAAGCAGAAGACGGCATACGAGAT**CTGATC**GTGACTGGAGTTCAGACGTGTGCTCTTCCGATCTNNNNNGTTGACATCTGCGGTAGCTG |
| rev_index10 | CAAGCAGAAGACGGCATACGAGAT**AAGCTA**GTGACTGGAGTTCAGACGTGTGCTCTTCCGATCTNNNNNGTTGACATCTGCGGTAGCTG |
| rev_index11 | CAAGCAGAAGACGGCATACGAGAT**GTAGCC**GTGACTGGAGTTCAGACGTGTGCTCTTCCGATCTNNNNNGTTGACATCTGCGGTAGCTG |
| rev_index12 | CAAGCAGAAGACGGCATACGAGAT**TACAAG**GTGACTGGAGTTCAGACGTGTGCTCTTCCGATCTNNNNNGTTGACATCTGCGGTAGCTG |
| F_adapter_GFPBC | AATGATACGGCGACCACCGAGATCTACACTCTTTCCCTACACGACGCTCTTCCGATCTNNNNNGGCCATCAAGCTTATCGATACC |
| R_adapter_GFPBC | CAAGCAGAAGACGGCATACGAGATCGTGATGTGACTGGAGTTCAGACGTGTGCTCTTCCGATCTNNNNNCTGATCAGCGAGCTCTAGTCG |
| Read 1 primer | ACACTCTTTCCCTACACGACGCTCTTCCGATCT |
| GFPBC_amp_Read2_1 | GTGACTGGAGTTCAGACGTGTGCTCTTCCGATCTCATGGTCCTGCTGGAGTTCGTGACC |
| GFPBC_amp_Read2_2 | GTGACTGGAGTTCAGACGTGTGCTCTTCCGATCTCGGGATCACTCTCGGCATGGACGAG |
| hRHO 1 Fw | AGGCCTTCGCAGCATTCTT |
| M.Fas. Rho R | AGTTCTCAACACCAGGAGCC |
| F_adapter_Rho | AATGATACGGCGACCACCGAGATCTACACTCTTTCCCTACACGACGCTCTTCCGATCTNNNNNTCTTCATGGTCTTCGGTGGC |
| R_adapter_MfusRHO_11 | CAAGCAGAAGACGGCATACGAGATGTAGCCGTGACTGGAGTTCAGACGTGTGCTCTTCCGATCTNNNNNCCTCCGCATTGCATCCTGT |
| R_adapter_MfusRHO_12 | CAAGCAGAAGACGGCATACGAGATTACAAGGTGACTGGAGTTCAGACGTGTGCTCTTCCGATCTNNNNNCCTCCGCATTGCATCCTGT |
| SaCas9 F1 | GTGGACCACATCATCCCCAG |
| SaCas9 R1 | GGTCTTGCTGATTCTGCCCT |
